# Antroquinonol reduces IL-1β production in macrophages through enhancing the DNA methylation of *Tlr4*

**DOI:** 10.3389/fnut.2025.1742566

**Published:** 2025-12-24

**Authors:** Peng Bin, Zhengyang Fu, Zixu Wang, Ifen Hung, Chunxue Liu, Wenjin Peng, Kaijun Wang, Jiahao Mo

**Affiliations:** 1 State Key Laboratory of Swine and Poultry Breeding Industry, College of Animal Science, South China Agricultural University, Guangzhou, China; 2 Anyou Biotechnology Group Co. LTD, Taicang, China; 3 Hunan Provincial Key Laboratory of the Traditional Chinese Medicine Agricultural Biogenomics, Changsha Medical University, Changsha, China; 4 College of Veterinary Medicine, Hunan Agricultural University, Changsha, China

**Keywords:** *Antrodia camphorata*, antroquinonol, macrophage, DNA methylation, IL-1β

## Abstract

**Introduction:**

Macrophages represent one of the most pivotal immune cells in the innate immune responses of weaned piglets. Emerging studies have revealed that numerous plant- or fungal-derived extracts significantly modulate macrophage functions. Antroquinonol (Antro), a characteristic triterpenoid compound isolated from *Antrodia camphorata*, has been extensively documented for its anti-inflammatory properties, but the precise mechanisms remain unclear.

**Methods:**

In this study, we established a macrophage polarization model *in vitro*, and evaluated the impact of Antro on inflammatory cytokine production in M1 macrophages. The expression of inflammatory pathway components was then measured to identify the specific targets regulated by Antro, and genetic manipulation approaches were further applied to validate these targets.

**Results:**

Antro enhances the enzymatic activities of DNA methyltransferases and facilitates DNA methylation-mediated suppression of *Tlr4* expression, thereby inhibiting NF-κB signaling, ultimately attenuating IL-1β production in macrophages.

**Conclusion:**

Our study elucidates a multi-pathway for the anti-inflammatory effects of Antro, significantly enriching the theoretical framework of natural product-mediated immunomodulation (particularly plant/fungal extracts). These findings provide critical scientific support for developing *A. camphorata*-derived fermentation products as novel feed additives to enhance immune function in swine production.

## 1 Introduction

As the key components of innate immunity, macrophages exhibit high heterogeneity and plasticity. They can polarize into classically activated (M1) or alternatively activated (M2) subtypes in response to environmental stimuli, thereby participating in immune regulation, tissue repair, and metabolic processes (1). During early inflammation, M1 macrophages secrete pro-inflammatory cytokines [e.g., interleukin (IL)-1β, IL-6, IL-12, tumor necrosis factor-α (TNF-α), and C-X-C Motif Chemokine Ligand 9 (CXCL9)] to recruit helper T cells and natural killer cells for pathogen clearance (2–4). However, persistent inflammation responses can damage intestinal epithelial cells and tissues in piglets, resulting in gastrointestinal dysfunction such as diarrhea (5, 6). Our previous studies shown that nutritional intervention, such as dietary γ-aminobutyric acid (GABA) supplementation, modulates intestinal innate immune function through influencing the intestinal macrophage polarization (7, 8). Mechanistically, GABA enhances succinate-flavin adenine dinucleotide (FAD)-lysine specific demethylase 1 (LSD1) signaling, which regulates the histone demethylation of *Bcl2l11* and *Dusp2* to inhibit the formation of the NLRP3-ASC-Caspase-1 complex in M1 polarized macrophages (8).

Intracellular signaling and metabolic pathways critically regulate the polarization of macrophages. For example, upon stimulation with lipopolysaccharide (LPS) and interferon-γ (IFN-γ), M1 macrophages rapidly reprogram the intracellular metabolic pathways, such as glycolysis, pentose phosphate pathway, and tricarboxylic acid cycle (9). This metabolic reprogramming enables metabolites (e.g., succinate and citrate) to stabilize hypoxia-inducible factor-1α (HIF-1α) and up-regulate pro-inflammatory cytokine expression (10, 11). In addition, the maintenance of macrophage polarization phenotypes and functions is governed by epigenetic regulation and post-translational modifications. For example, the DNA methylation of Galectin-8 mediates macrophage autophagy and aggravates the inflammation through MAPK/mTOR pathway (12). Our previous study also demonstrated that phosphoglycerate dehydrogenase (PHGDH)-mediated NAD^+^ accumulation facilitates the transcription of *Tlr4* through H3K9/27 acetylation, thereby sustaining *Il1b* expression in macrophage (13).

**Figure 1:**
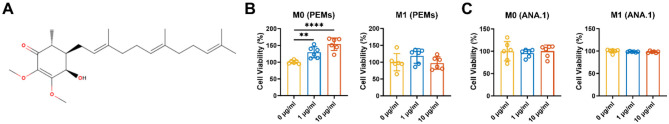
Effects of Antro on macrophage viability. **(A)** The chemical structure of Antro; **(B)** Cell viability of PEMs treated with different concentrations of Antro (*n* = 6); **(C)** Cell viability in ANA.1 treated with different concentrations of Antro (*n* = 6). Data were analyzed by one-way ANOVA with Dunnett's test and presented as mean ± SD. **P < 0.001, ****P < 0.0001.

*Antrodia camphorata* is a rare medicinal and edible fungus with detoxification, hepatoprotective, and anticancer properties (14–16). However, natural *A. camphorata* resources remain scarce due to its extended growth cycle and exclusive host specificity (*Camphora kanahirae*), it is primarily utilized as a therapeutic agent in the management of hepatometabolic disorders. With the recent advancements in artificial cultivation, the fermentation products of *A. camphorata* have been increasingly utilized in livestock and poultry farming. Previous studies have demonstrated that supplementation of *A. camphorata* fermentation products improves the organism antioxidant capacity and anti-inflammatory capacity in weaned piglets and laying hens (17, 18). Our previous study also demonstrated that the *A. camphorata* fermentation products decreased the levels of inflammatory cytokines in piglets and Largemouth bass. However, the specific bioactive components and molecular mechanisms by which *A. camphorata* modulates inflammatory cytokine production remain incompletely characterized, which limits its optimized application serves as food or feed additives. Antroquinonol (Antro) is a unique tetracyclic triterpenoid compound (Figure 1A) endogenous to *A. camphorata* that confers both anticancer and anti-inflammatory properties (19). Antro directly binds to phosphatidylinositol 3-kinase (PI3K) to inhibit the activation of serine/threonine kinase AKT, suppressing the expression of the oncogenic transcription factor β-catenin and downstream target genes (20). In the animal models, previous studies demonstrated that Antro decreased systemic IL-1β and TNF-α levels, confirming its anti-inflammatory activity (21). However, the precise molecular mechanisms underlying these effects remain to be elucidated.

In this study, we evidenced Antro elevates the enzymatic activity of DNA to promotes DNA methylation at the promoter region of *Tlr4* and suppress nuclear factor kappa-B (NF-κB) signaling pathways, ultimately decreasing the IL-1β production in M1 macrophages. Our results elucidate the molecular mechanism by which Antro regulates the macrophage functions, and provided a theoretical basis for the utilization of *A. camphorata*-derived products as novel immunoregulatory food or feed additives.

## 2 Materials and methods

### 2.1 Cell lines and cell culture

The murine macrophage cell line ANA.1 (National Infrastructure of Cell Line Resource, 3101MOUGNM 2) was generously provided by Professor Yuexia Liao (Yangzhou University). Cells were cultured at 37 °C with 5% CO_2_ in DMEM (Gibco, USA) supplemented with 10% fetal bovine serum (ExCell Bio, China) and 1% penicillin-streptomycin (Gibco, USA). For M1 polarization, cells were first treated with Antro and then simultaneously stimulated with 1 μg/ml LPS from *Escherichia coli* O55:B5 (Sigma, USA, catalog number L2880) and 20 ng/ml IFN-γ (Proteintech, USA) for 6 h. The medium was aspirated following stimulation, and cells were washed thoroughly three times with warm PBS to remove residual LPS. Fresh complete medium containing the same concentration of Antro was then added, and both cells and supernatants were harvested for the further analysis after 6 h treatment.

### 2.2 Isolation of antroquinonol

Antro used in this study was extracted and purified from the fermented products of *A. camphorata* by Wuxi AppTec Co., Ltd. The culture conditions for *A. camphorata* and the extraction protocol for Antro followed a previously established method (22). Following extraction and purification, the purity of Antro was determined by High Performance Liquid Chromatography (HPLC), and its structure was verified by Nuclear Magnetic Resonance (NMR) and Mass Spectrometry (MS) analyses.

### 2.3 Primary peritoneal macrophages isolation

The protocol of primary peritoneal exudate macrophages (PEMs) have been described previously (23). Briefly, murine PEMs were isolated from ICR mice (6 weeks) 3 days after intraperitoneal injection of 4% thioglycolate (Sigma, Germany). Cells were harvested by peritoneal lavage with phosphate-buffered saline (PBS, Gibco, USA), lysed with red blood cell lysis buffer (CWBIO, China), and cultured in DMEM medium (supplemented with 10% FBS and 1% penicillin-streptomycin). Macrophage purity was determined to be 94.6%. The culture conditions and handling procedures for PEMs are identical to those for established cell lines.

### 2.4 Cell counting kit 8 (CCK8) assay

Cell viability was monitored using CCK8 kit (Dojindo, Japan) according to the protocol provide by manufacturers. Briefly, M0 or M1 macrophages (1 × 10^5^ cells/well) were seeded in 96-well plates and treated with varying concentrations of Antro (extracted by WuXi AppTec, China). After treatment, 10 μl of CCK-8 solution was added to each well, followed by incubation at 37 °C for 1 h. Absorbance was then measured at 450 nm using a microplate reader (Varioskan LUX, Thermo Fisher Scientific, USA).

### 2.5 Enzyme-linked immunosorbent assay (ELISA)

Cell supernatant was harvested and centrifuged at 1,000 g for 5 min, and the levels of pro-inflammatory cytokines were measured using ELISA (CSB-E08054m, Cusabio, China) according to the manufacturer's instruction.

### 2.6 Quantitative polymerase chain reaction (qPCR)

Total RNA was isolated with RNA Purification Kit (EZ Bioscience, USA) and reverse transcribed into cDNA using Color Reverse Transcription Kit (EZ Bioscience, USA). qPCR was then performed using SYBR Green qPCR Master Mix (EZ Bioscience, USA) on QuantStudio 6 Pro system (Thermo Fisher Scientific, USA). Primer sequences used in this study are listed in Table 1. Data were normalized to β-actin as the reference gene and analyzed using the 2^−Δ*ΔCT*^ method.

**Table 1:** List of qPCR primers used in this study.

**Primer**	**Primer sequence (5^′^-3^′^)**
Mus *β-actin* F	GTGACGTTGACATCCGTAAAGA
Mus *β-actin* R	GCCGGACTCATCGTACTCC
Mus *Il1b* F	GAAATGCCACCTTTTGACAGTG
Mus *Il1b* R	TGGATGCTCTCATCAGGACAG
Mus *Tnfa* F	CAGGCGGTGCCTATGTCTC
Mus *Tnfa* R	CGATCACCCCGAAGTTCAGTAG
Mus*Tlr4* F	GCCTTTCAGGGAATTAAGCTCC
Mus *Tlr4n* R	GATCAACCGATGGACGTGTAAA
Mus *Myd88* F	TCATGTTCTCCATACCCTTGGT
Mus *Myd88* R	AAACTGCGAGTGGGGTCAG
Mus *Trif* F	AACCTCCACATCCCCTGTTTT
Mus *Trif* R	GCCCTGGCATGGATAACCA
Mus *Asc* F	GGACGGAGTGCTGGATGCTTTG
Mus *Asc* R	TGAGGCTGCAGTTGTCTAATTCC
Mus *Nlrp3* F	CCTTGGAGACACAGGACTCA
Mus *Nlrp3* R	TGAGGCTGCAGTTGTCTAATTCC
Mus *Caspase 1* F	CCAGGAGGGAATATGTGGGAC
Mus *Caspase 1* R	ACTCCTTGTTTCTCTCCACGG
Mus *Dnmt1* F	CCGTGGCTACGAGGAGAAC
Mus *Dnmt1* R	TTGGGTTTCCGTTTAGTGGGG
Mus *Dnmt3a* F	GATGAGCCTGAGTATGAGGATGG
Mus *Dnmt3a* R	CAAGACACAATTCGGCCTGG
Mus *Dnmt3b* F	CGTTAATGGGAACTTCAGTGACC
Mus *Dnmt3b* R	CTGCGTGTAATTCAGAAGGCT
Mus *Dnmt3l* F	TACGAAGTCAAAGTGAACCGAC
Mus *Dnmt3l* R	ACAAGGGGTGCCGAGTGTA

### 2.7 Immunoblotting

Cells were lysed using RIPA Lysis Buffer (Beyotime, China), and protein concentration was quantified with the enhanced BCA Protein Assay Kit (Beyotime, China) prior to separation via SDS-PAGE. Resolved proteins were electrophoretically transferred to polyvinylidene difluoride (PVDF) membranes. Membranes were blocked with Tris-Tween-buffered saline buffer (TBST) and incubated with primary antibodies at 4 °C overnight. Following TBST washes, membranes were probed with HRP-conjugated secondary antibodies at room temperature for 90 min. Chemiluminescent substrate was applied, and signals were captured by X-ray film exposure. After film development, band intensities were quantified using Quantity One software (v4.6.6).

### 2.8 Global DNA methylation assay

Genomic DNA was extracted using the TIANamp Genomic DNA Kit (Tiangen, China), and global DNA methylation levels were quantified from purified DNA samples using the MethylFlash Global DNA Methylation (5-mC) ELISA Kit (EpigenTek, USA) according to the manufacturer's protocol.

### 2.9 Quantitative methylation specific PCR (qMSP)

DNA (3 μg) was diluted to 50 μl with sterile double-distilled water and treated with NaOH to a final concentration of 0.2 M, followed by incubation at 42 °C for 30 min. Subsequently, 30 μl of 10 mM hydroquinone, 520 μl of 3 M sodium bisulfite (pH 5.0), and 200 μl of paraffin oil were added. The mixture was incubated at 50 °C for 16 h in the dark. Bisulfite-converted DNA was purified by EZ-10 Column DNA Purification Kit (Sangon Biotech, China), and qPCR was performed with the Methylamp MS-qPCR Fast Kit (EpigenTek, USA). Primer sequences are listed in Table 2.

**Table 2:** List of MSP primers used in this study.

**Primer**	**Primer sequence (5^′^-3^′^)**
Meth *Tlr4* F	TTGAGAATTGAGAATTGTAGAAGGTATT
Meth *Tlr4* R	AACCCAAAATAAAAATATCATCAATTAC

### 2.10 DNA methyltransferases activity

Nuclear proteins were extracted using the EpiQuik Nuclear Extraction Kit I (Epigentek USA). The extracted nuclear protein (10 μg) was assayed for DNA methyltransferase (DNMT) activity using the EpiQuik DNMT Activity/Inhibition ELISA Kit (Epigentek, USA) according to the manufacturer's protocol.

### 2.11 Molecular docking analysis

Molecular docking of Antro to the DNMT protein was performed using AutoDock Vina, with all water molecules excluded from the simulation. The resulting ligand-protein interactions were visualized and analyzed using a two-dimensional diagram generated with Discovery Studio 4.5.

### 2.12 Statistical analyses

Data were analyzed using GraphPad Prism (V9.5) and expressed as mean ± SD or mean ± SEM. Multi-group comparisons were evaluated by one-way ANOVA with Dunnett's test. For two-group comparisons, statistical tests were selected as follows: unpaired *t*-test for normally distributed data with equal variances; Welch's *t*-test for normally distributed data with unequal variances; and Mann-Whitney *U*-test for non-normally distributed data. The differences were considered significant at *P* < 0.05.

## 3 Results

### 3.1 Antro did not affect cell viability of macrophage

Given the unknown effects of Antro on macrophage activity, we employed a cell viability assay to evaluate its impact on primary elicited macrophages (PEMs; M0 and M1 phenotypes) and the ANA.1 cell line. Results indicated that Antro treatment significantly increased the cell viability of peritoneal M0 macrophages (*P* < 0.05) but had no significant effect on peritoneal M1 macrophages (Figure 1B). Furthermore, Antro did not significantly alter ANA.1 cell viability (Figure 1C). Collectively, these data demonstrate that Antro did not suppress the cell viability of tested macrophages.

### 3.2 Antro inhibited IL-1β production in M1 macrophages

**Figure 2:**
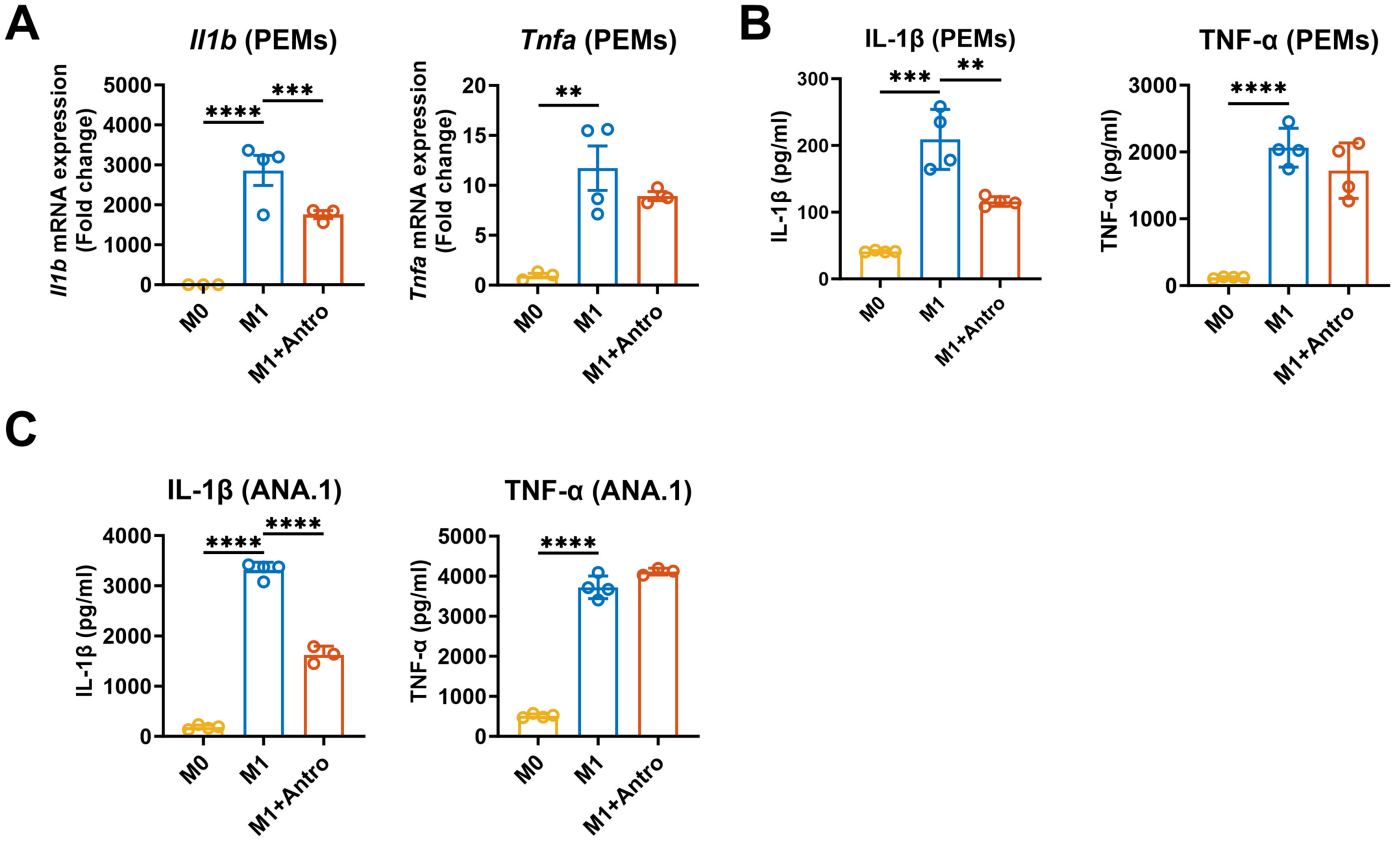
Antro reduces IL-1β production and *Il1b* expression in M1 macrophages. **(A)** Secretion levels of IL-1β and TNF-α in PEMs treated with or without 1 μg/ml Antro for 12 h (*n* = 4); **(B)** mRNA expression of *Il1b* and *Tnfa* in PEMs treated with or without 1 μg/ml Antro for 12 h (*n* = 4); **(C)** Secretion levels of IL-1β and TNF-α in ANA.1 cells treated with or without 1 μg/ml Antro for 12 h (*n* = 4). Data between two groups were analyzed by an unpaired *t*-test and presented as mean ± SD or mean ± SEM (mRNA data). **P < 0.01, ***P < 0.001, ****P < 0.0001.

Based on cell viability results, a concentration of 1 μg/ml Antro was selected for subsequent experiments. To further assess the impact of Antro on macrophage inflammatory responses, we next measured characteristic M1 macrophage cytokines (IL-1β and TNF-α) for 12h after Antro treatment. Results showed that Antro significantly reduced *Il1b* gene expression and IL-1β protein production in primary macrophages (*P* < 0.05) but did not affect *Tnfa* expression or TNF-α production (Figures 2A, B), and the similar results was also found in ANA.1 cell line (Figure 2C). Together, these findings demonstrate that Antro selectively inhibits IL-1β production in M1-polarized macrophages without altering TNF-α production.

### 3.3 Antro suppressed IL-1β-related inflammatory pathways in M1 macrophages

**Figure 3:**
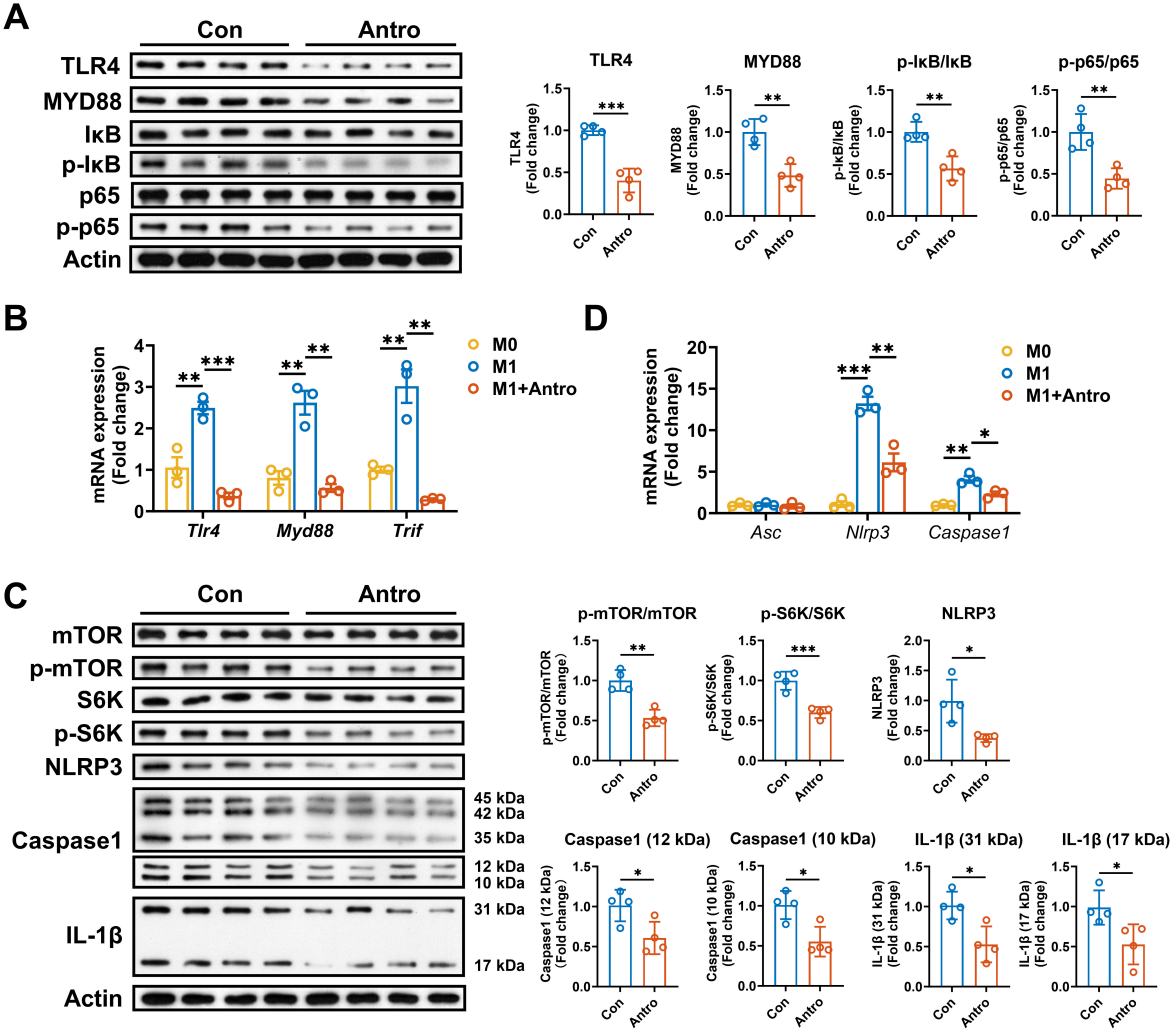
Antro suppresses pro-inflammatory signaling pathways in ANA.1 cells. **(A)** Protein levels of NF-κB pathway components in M1 macrophages treated with or without Antro (*n* = 4); **(B)** mRNA expression of NF-κB pathway components in M1 macrophages treated with Antro or without Antro (*n* = 3); **(C)** Protein levels of mTOR and inflammasome pathway components in M1 macrophages treated with Antro or vehicle control (*n* = 3). **(D)** mRNA expression of genes related to the NLRP3 inflammasome in M1 macrophages treated with Antro or without Antro (*n* = 4). Data between two groups were analyzed by an unpaired *t*-test and presented as mean ± SD or mean ± SEM (mRNA data). *P < 0.05, **P < 0.01, ***P < 0.001.

IL-1β production is regulated by signaling pathways including NF-κB, mammalian target of rapamycin (mTOR), and inflammasome pathways (24–26). To investigate the mechanism by which Antro inhibits IL-1β production in M1 macrophages, we analyzed the expression of those key signaling components. Antro significantly reduced TLR4 and Myd88 protein expression and decreased phosphorylation ratios of IκB (p-IκB/IκB) and p65 (p-p65/p65; *P* < 0.05, Figure 3A). Correspondingly, *Tlr4, Myd88*, and *Trif* mRNA levels were also downregulated (*P* < 0.05, Figure 3B). In addition to NF-κB pathway, Antro reduced p-mTOR/mTOR and p-S6K/S6K ratios and NLRP3 and Caspase1 protein expression (*P* < 0.05, Figure 3C), and downregulated the mRNA expression of *Nlrp3* and Caspase1 (*P* < 0.05, Figure 3D). These results demonstrate that Antro inhibits IL-1β production through suppression of NF-κB signaling pathway.

### 3.4 Antro inhibited IL-1β production through decreasing the Tlr4 mRNA expression

**Figure 4:**
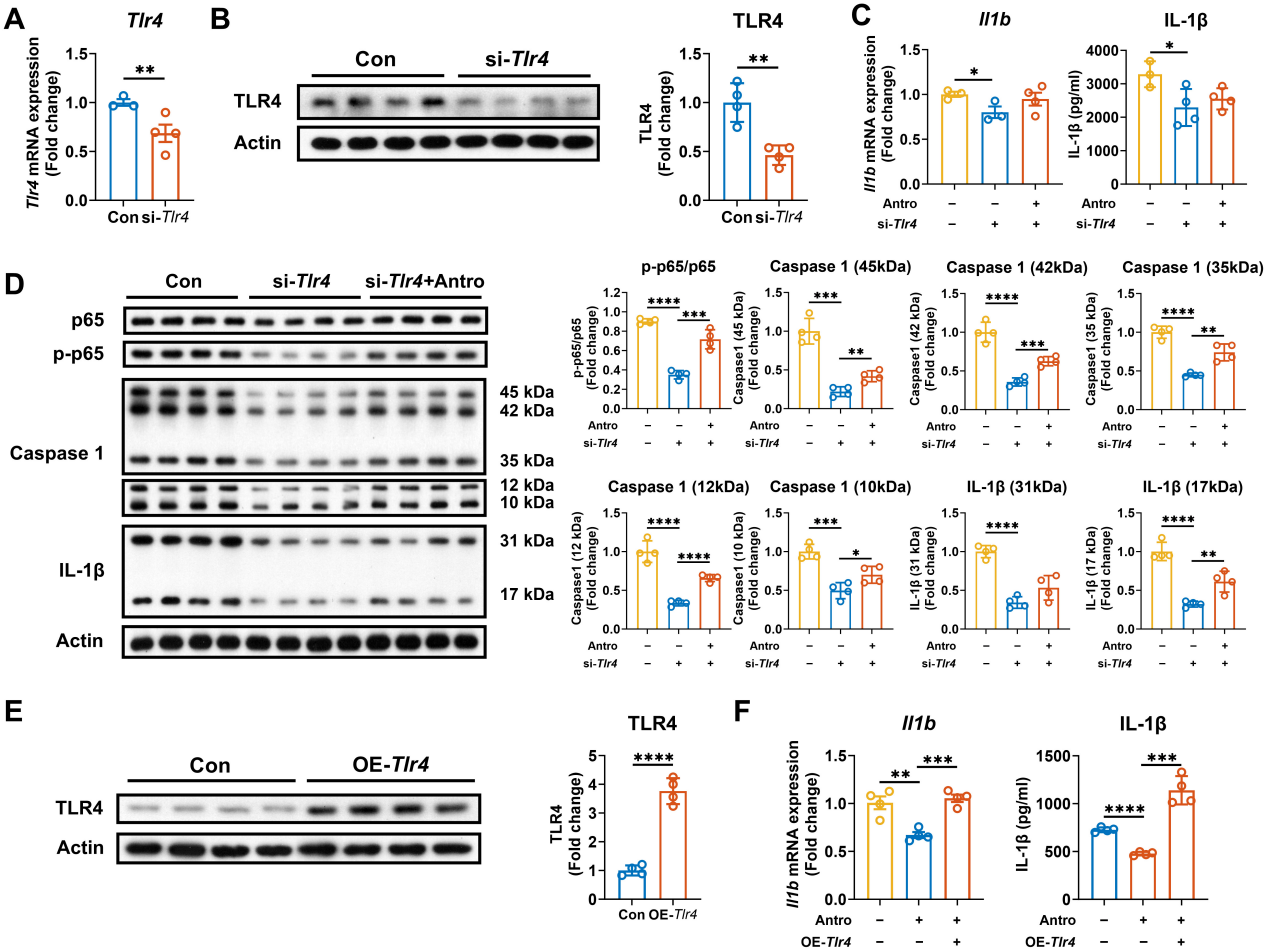
Antro reduces IL-1β production by inhibiting *Tlr4* expression in ANA.1 cells. **(A)** mRNA expression of *Tlr4* expression in M1 macrophages treated with or without si-*Tlr4* (*n* = 4); **(B)** Protein level of TLR4 in M1 macrophages treated with or without si-*Tlr4* (*n* = 4); **(C)** mRNA expression and protein expression of IL-1β in *Tlr4* silenced-M1 macrophages treated with or without Antro (*n* = 4); **(D)** Protein levels of NF-κB pathway components in *Tlr4* silenced-M1 macrophages treated with or without Antro (*n* = 4); **(E)** TLR4 protein level in *Tlr4*-overexpressing M1 macrophages or control (*n* = 4); **(F)** mRNA and protein expression of IL-1β in *Tlr4*-overexpressing M1 macrophages treated with or without Antro (*n* = 4). Data between two groups were analyzed by an unpaired *t*-test and presented as mean ± SD or mean ± SEM (mRNA data). *P < 0.05, **P < 0.01, ***P < 0.001, ****P < 0.0001.

As NF-κB functions upstream of mTOR and inflammasome pathways, we speculated that Antro reduces IL-1β production in M1 macrophages primarily through suppressing the mRNA expression of *Tlr4* (the most upstream initiator). To verify this, we employed a genetic approach to silence *Tlr4* expression by siRNA (Figures 4A, B). *Tlr4* silencing abrogated the inhibitory effects of Antro on *Il1b* expression and IL-1β production in M1 macrophages (*P* < 0.05, Figure 4C). Furthermore, it eliminated the suppression of Antro on the p-p65/p65 ratio and protein levels of Caspase1 and IL-1β (*P* < 0.05, Figure 4D). To further validate the critical role of *Tlr4* in Antro-induced IL-1β reduction, we constructed and transfected the *Tlr4* overexpression plasmids into M1 macrophages (Figure 4E). As expected, *Tlr4* overexpression rescued the inhibitory effects of Antro on *Il1b* expression and IL-1β production in M1 macrophages (Figure 4F). Collectively, these results demonstrated that Antro inhibits IL-1β production in M1 macrophages through downregulating the mRNA expression of *Tlr4*.

### 3.5 Antro downregulated Tlr4 expression by DNA methylation

**Figure 5:**
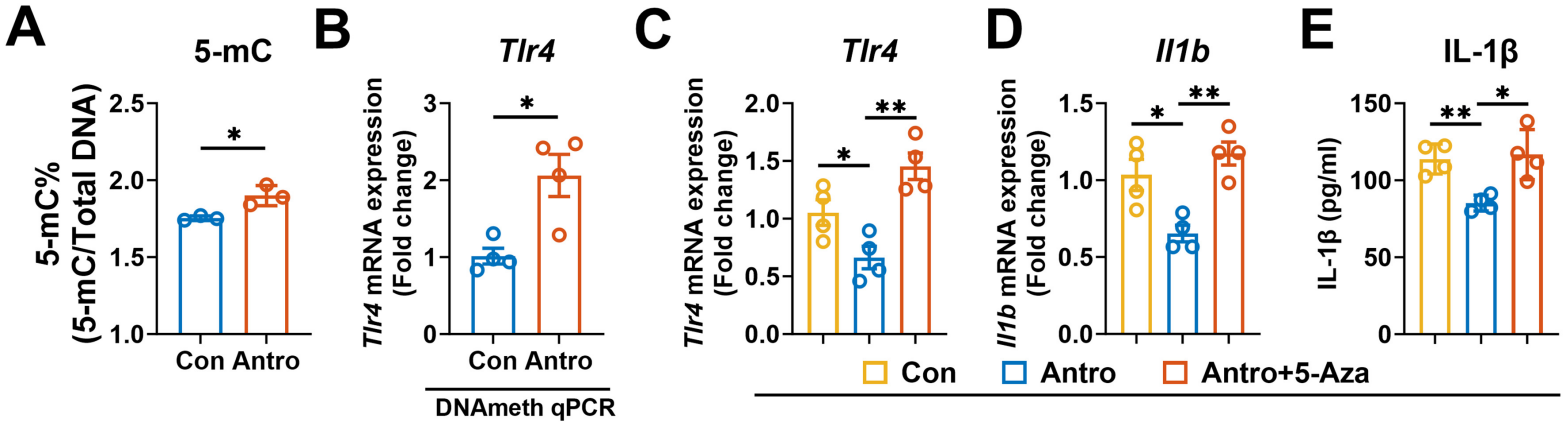
Antro suppresses *Tlr4* expression through enhancing DNA methylation in ANA.1 cells. **(A)** Global DNA methylation in M1 macrophages treated with or without Antro (*n* = 4); **(B)** *Tlr4* methylation in M1 macrophages treated with or without Antro (*n* = 6); **(C–E)** *Tlr4* **(C)** and *Il1b* **(D)** expression and IL-1β level **(E)** in Antro-treated M1 macrophages with or without 5-Aza (*n* = 4). Data between two groups were analyzed by an unpaired *t*-test and presented as mean ± SD or mean ± SEM (mRNA data). *P < 0.05, **P < 0.01.

Previous studies have demonstrated that Antroquinonol D, a ubiquinone derivative isolated from *A. camphorata*, functions as a DNA methyltransferase regulator (27). Given the structural similarity between Antroquinonol D and Antro, we speculated Antro might regulate DNA methylation to modulate *Tlr4* expression. Consequently, we measured global DNA methylation in M1 macrophages treated with Antro. The results showed that Antro significantly increased the percentage of 5-methylcytosine (5-mC; *P* < 0.05, Figure 5A), indicating enhanced global DNA methylation. To assess whether Antro increased the DNA methylation of *Tlr4*, we performed quantitative methylation specific PCR and observed increased DNA methylation of *Tlr4* (*P* < 0.05, Figure 5B). Subsequently, we treated M1 macrophages with the DNA methyltransferase inhibitor 5-aza-cytidine hydrate (5-Aza), which abolished Antro-mediated suppression of *Tlr4* and *Il1b* expression, as well as IL-1β production (*P* < 0.05, Figures 5C–E). Collectively, these results indicate that Antro suppresses *Tlr4* expression through promoting the DNA methylation of *Tlr4*.

### 3.6 Antro increased DNMT activity by directly binding to DNA methyltransferases

**Figure 6:**
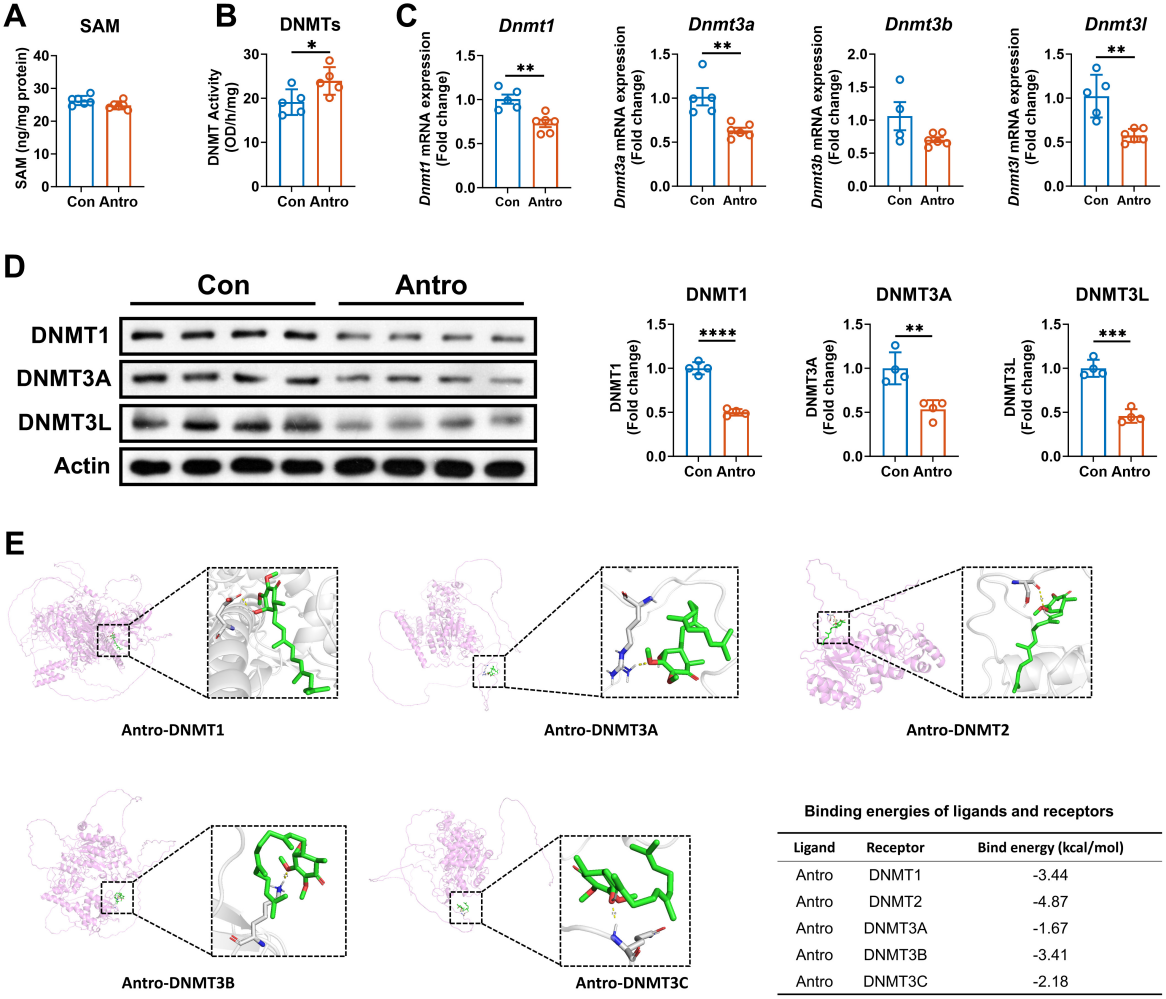
Antro enhances the enzymic activities of DNMTs in ANA.1 cells. **(A)** SAM level in M1 macrophages treated with or without Antro (*n* = 6); **(B)** DNMT enzymic activities in M1 macrophages treated with or without Antro (*n* = 6); **(C, D)** mRNA **(C)** and protein **(D)** expression in M1 macrophages treated with or without Antro (*n* = 4). **(E)** Docking results and binding energies of Antro with DNA methyltransferases. Data between two groups were analyzed by an unpaired *t*-test and presented as mean ± SD (protein data) or mean ± SEM (mRNA data). *P < 0.05, **P < 0.01, ***P < 0.001, ****P < 0.0001.

In the catalysis of DNMTs, DNA undergoes methylation to alter chromatin structure or DNA conformation through the transfer of methyl groups from *S*-adenosylmethionine (SAM), thereby regulating the gene expression (28, 29). To clarify the mechanism by which Antro regulates DNA methylation, we analyzed the impact of Antro on substrate level and enzyme activity. Antro did not alter cellular SAM levels (Figure 6A) but significantly increased DNMT activity (*P* < 0.05, Figure 6B), indicating that Antro regulates DNA methylation by enhancing DNMT activities. We next investigated how Antro affects DNA methyltransferase activity at the mRNA and protein expression level. Interestingly, the results showed that Antro decreased the mRNA expression of DNMT-encoding genes, including *Dnmt1, Dnmt3a*, and *Dnmt3l* (*P* < 0.05, Figure 6C). In addition, Antro reduced the protein expression of DNMT1, DNMT3A, and DNMT3L (*P* < 0.05, Figure 6D). These findings indicate that Antro increases DNMT activity independently of transcriptional or translational processes, likely through direct binding to DNMTs to enhance their enzymatic activity. To validate the potential binding interactions between Antro and DNMTs, we performed molecular docking analyses, which demonstrated strong binding affinities between Antro and five target DNMTs (Figure 6E). These results provide structural evidence for direct molecular interactions, supporting a mechanism where Antro boosts DNMT activity post-translationally.

## 4 Discussion

Our prior unpublished data indicate that *A. camphorata* fermentation products reduced mortality in pigs during outbreaks of macrophage-associated viral infections (e.g., porcine reproductive and respiratory syndrome), indicating its regulatory role in macrophage function. In addition, we also demonstrated that supplementation of *A. camphorata* fermentation products decreased the levels of inflammatory cytokines in piglets and Largemouth bass. *A. camphorata* contains over 200 bioactive compounds, including triterpenoids, polysaccharides, and benzene derivatives. Among these, Antro is notable for its unique furocyclohexenone structure, which confers potent antioxidant and immunomodulatory properties (30, 31). Therefore, we selected Antro to investigate its regulatory role on macrophage function and the underlying molecular mechanisms. In this study, we found that Antro may directly bind to DNMTs to enhance their enzymes activities, thereby promoting the DNA methylation of *Tlr4*. This mechanism inhibits the activation of NF-κB, subsequently reducing IL-1β production in activated M1 macrophages. Notably, Antro did not affect TNF-α production in M1 macrophages, and similar findings were observed in breast cancer cell lines (32). The selective regulation of IL-1β may be attributed to the distinct signaling pathways involved. Our results indicate that Antro suppresses the initial priming signal (TLR4/NF-κB) via DNA methylation of *Tlr4*, thereby attenuating both pro-IL-1β synthesis and the expression of genes related to the NLRP3 inflammasome. This suppression inhibits both the transcription of pro-IL-1β and its cleavage, and it exerts a more pronounced effect on IL-1β production. This pattern of selective IL-1β regulation is consistent with previous studies (8, 33, 34). Interestingly, a separate study reported that Antro reduced circulating TNF-α levels in mice (21). These results suggest that Antro may selectively modulate TNF-α production in specific cell types (35, 36), such as lymphocytes, dendritic cells or mast cells.

IL-1β functions as the central effector molecule in inflammatory response, with its production modulated through multistep cascade reactions. TLRs recognizes pathogen-associated molecular patterns, thereby activating NF-κB signaling pathway to induce the expression of pro-IL-1β. Signal molecules such as adenosine triphosphate (ATP) activate the NLRP3 inflammasome, which recruits Caspase1 to cleave pro-IL-1β and generates mature IL-1β (37, 38). In this study, we found that Antro inhibits the phosphorylation of p65, indicating that its primary anti-inflammatory mechanism involves suppression of the TLR4-NF-κB pathway. This suppression impairs p65 nuclear translocation and the subsequent transcription of inflammatory mediators (39). By targeting this upstream signaling hub, Antro may effectively modulates the cellular readiness for inflammasome assembly and pro-IL-1β production.

Although Antro has been shown to inhibit NF-κB signaling pathway activation in previous studies, the specific target genes or proteins remains incompletely elucidated. TLR4 is a crucial pattern recognition receptor that triggers signal cascades and activates the downstream NF-κB signaling pathway (40). Our findings demonstrated that Antro decreased the mRNA expression of *Tlr4* and inhibited the activation of the NF-κB signaling. However, mRNA expression is regulated by multiple mechanisms, including epigenetic modifications or alterations in transcription factor activity (41, 42). Previous studies suggested that antroquinonol D acts as a DNMT1 inhibitor to modulate DNA methylation (27), we hypothesize that Antro may regulate *Tlr4* expression via DNA methylation-mediated mechanisms. DNA methylation inhibits gene expression via two mechanisms: (1) direct interference with transcription factor binding to gene promoters; (2) recruitment of transcriptional repressors through 5-mC-binding proteins (43, 44). In this study, we demonstrated that Antro enhances the DNA methylation of *Tlr4*, thereby downregulating its expression in M1 macrophages. This finding indicates that Antro directly targets upstream inflammatory signaling pathways, potently suppressing the inflammatory cascade at an earlier stage.

The DNA methylation process is modulated by DNMT activity and substrate (SAM) availability. Notably, previous studies have shown that an Antro analog binds to the catalytic domain of DNMT1, which competes with SAM for the same binding pocket within the DNMT1 enzyme, thereby inhibiting DNMT1 enzymatic activity (27). In the current study, our findings indicate that Antro does not influence SAM levels or DNMT expression in macrophages, yet it can enhance the enzymatic activity of DNMTs. Certain small molecules, termed allosteric effectors, bind to specific enzyme to induce conformational changes that markedly enhance catalytic activity. For instance, MK-0941 is a small-molecule glucokinase activator that enhances catalytic activity by binding to the enzyme and stabilizing a conformational state with a substantially higher affinity for glucose (45). The increase of these activators results in remarkably high catalytic efficiency per enzyme molecule, even with limited enzyme abundance. Our molecular docking analyses demonstrated that Antro exhibits binding affinity for five target DNMTs, we therefore speculate that Antro may directly bind to DNMTs to enhance their catalytic function, although its precise binding mode and site remain unidentified.

## 5 Conclusion

In summary, we elucidate the mechanism by which Antro regulates the IL-1β production in M1 macrophages. Antro enhances DNMT enzymic activity and promotes the DNA methylation of *Tlr4*. This epigenetic modification inhibits NF-κB activation and reduces IL-1β production. These findings expand the theoretical framework for immune modulation by natural products (plant/fungal extracts), and provide a foundation for developing novel food or feed additives derived from *A. camphorata* to elevate immune function.

## 6 Limitations of this study

We observed that Antro reduces IL-1β production by promoting DNA methylation of *Tlr4* in M1 macrophages. It should be noted that the mechanistic insights in this study were primarily derived from *in vitro* experiments, future studies to validate the immune-modulatory effects of Antro in murine models of inflammatory disease will be essential for establishing its relevance as a practical food or feed additive.

## Data Availability

The datasets presented in this study can be found in online repositories. The names of the repository/repositories and accession number(s) can be found in the article/supplementary material.
